# Brain delivery of erythropoietin results in a significant reduction of brain Aβ and spatial memory deficits in the APP knock‐in mouse model

**DOI:** 10.1002/alz.090625

**Published:** 2025-01-09

**Authors:** Rudy Tieu Chang, Devaraj V. Chandrashekar, Grace‐Ann Chuli Roules, Emi Iwasaki, Nataraj Jagadeesan, Rachita K. Sumbria

**Affiliations:** ^1^ School of Pharmacy, Chapman University, Irvine, CA USA; ^2^ Schmid College of Science and Technology, Orange, CA USA; ^3^ University of California, Irvine, Irvine, CA USA

## Abstract

**Background:**

Although novel treatments for Alzheimer’s disease (AD) have begun to show modest therapeutic effects, agents that target hallmark AD pathology and offer neuroprotection are desired. Erythropoietin (EPO) is a glycoprotein hormone with neuroprotective effects but is faced with challenges including limited brain uptake and increased hematopoietic side effects with long‐term dosing. Therefore, EPO has been modified and bound to a chimeric transferrin receptor monoclonal antibody (cTfRMAb); the latter shuttles EPO past the blood‐brain barrier (BBB) into brain parenchyma and reduces its plasma exposure and potential for side effects. Our study sought to characterize the safety and pharmacokinetics (PK) of modified EPO following chronic dosing in healthy mice, and then utilize the optimized dose in mitigating hallmark AD pathologies in APP‐SAA knock‐in (KI) mice, a model that recapitulates Aβ pathology in the absence of APP overexpression, *in vivo*.

**Methods:**

For the PK and safety study, a multidose design was employed with 10‐week‐old C57BL/6 male mice (n = 4‐5/dose) receiving doses ranging from 1‐ to 20‐mg/kg SQ for 4 weeks, aimed to evaluate the dose‐dependent plasma concentrations, and metabolic and hematologic safety of the modified EPO. The dose that resulted in the highest safety and sustained plasma exposure was dosed SQ to 5.5‐month‐old male APP‐SAA KI mice (n = 6) for 14 weeks. Control APP‐SAA KI mice (n = 5) received vehicle. The effect of modified EPO on Aβ load by immunoassays, and spatial memory via Y‐maze test, were assessed.

**Results:**

The 1mg/kg dose resulted in no adverse effects and sustained plasma exposure which are conducive to longitudinal dosing. APP‐SAA KI mice treated with the modified EPO had a remarkable (70‐80%, p<0.001) reduction in 6E10‐positive‐Aβ plaque area and number in the brain. Aggregated Aβ measured by ELISA was also significantly lower (p<0.05) with modified EPO treatment. Modified EPO increased the discrimination index for the novel arm (p<0.05), suggesting an improvement in spatial memory recall of the reinforced arm of the maze.

**Conclusions:**

These findings provide essential data for dose optimization for longitudinal studies using cTfRMAb‐based therapeutics, and specifically modified EPO used herein, and illustrate the therapeutic potential of the brain‐penetrating cTfRMAb‐EPO in a novel AD mouse model devoid of APP overexpression.

